# Direct Comparison of Therapeutic Effects on Diabetic Polyneuropathy between Transplantation of Dental Pulp Stem Cells and Administration of Dental Pulp Stem Cell-Secreted Factors

**DOI:** 10.3390/ijms21176064

**Published:** 2020-08-23

**Authors:** Saki Kanada, Eriko Makino, Nobuhisa Nakamura, Megumi Miyabe, Mizuho Ito, Masaki Hata, Taisuke Yamauchi, Noritaka Sawada, Shun Kondo, Tomokazu Saiki, Tomomi Minato, Ken Miyazawa, Shigemi Goto, Tatsuaki Matsubara, Keiko Naruse

**Affiliations:** 1 Department of Orthodontics, School of Dentistry, Aichi Gakuin University, Nagoya 464-8651, Japan; ag173d06@dpc.agu.ac.jp (S.K.); eriko@dpc.agu.ac.jp (E.M.); ag193D19@dpc.agu.ac.jp (T.Y.); miyaken@dpc.agu.ac.jp (K.M.); shig@dpc.agu.ac.jp (S.G.); 2 Department of Internal Medicine, School of Dentistry, Aichi Gakuin University, Nagoya 464-8651, Japan; nnaka@dpc.agu.ac.jp (N.N.); mmiyabe@dpc.agu.ac.jp (M.M.); i-mizuho@dpc.agu.ac.jp (M.I.); matt@dpc.agu.ac.jp (T.M.); 3 Department of Removable Prosthodontics, School of Dentistry, Aichi Gakuin University, Nagoya 464-8651, Japan; hata@dpc.agu.ac.jp; 4 Department of Periodontology, School of Dentistry, Aichi Gakuin University, Nagoya 464-8651, Japan; ag173D11@dpc.agu.ac.jp (N.S.); ag193d07@dpc.agu.ac.jp (S.K.); 5 Department of Pharmacy, Aichi Gakuin University, Dental Hospital, Nagoya 464-8651, Japan; saiki@dpc.agu.ac.jp; 6 Department of Clinical Laboratory, Aichi Gakuin University, Dental Hospital, Nagoya 464-8651, Japan; minato19@dpc.agu.ac.jp

**Keywords:** dental pulp stem cell, diabetic polyneuropathy, secreted factor, transplantation, regenerative medicine

## Abstract

Stem cell transplantation is a potential novel therapy for diabetic polyneuropathy. Dental pulp stem cells (DPSCs) are attractive stem cell sources because DPSCs can be isolated from extracted teeth and cryopreserved while retaining viability. In this study, we directly compared the efficacy of the transplantation of DPSCs and the administration of the secreted factors from DPSCs (DPSC-SFs) on diabetic polyneuropathy. Eight weeks after streptozotocin injection, DPSCs (1.0 × 10^6^ cells/rat) or DPSC-SFs (1.0 mL/rat) were administered into the unilateral hindlimb skeletal muscles of diabetic Sprague–Dawley rats. DPSC transplantation and DPSC-SF administration did not affect blood glucose levels and body weights in the diabetic rats. Both DPSC transplantation and DPSC-SF administration significantly ameliorated sciatic nerve conduction velocity and sciatic nerve blood flow, accompanied by increases in muscle bundle size, vascular density in the skeletal muscles and intraepidermal nerve fiber density in the diabetic rats, while there was no difference between the results for DPSCs and DPSC-SFs. These results suggest that the efficacy of both DPSC transplantation and DPSC-SF administration for diabetic polyneuropathy four weeks after transplantation/administration was mainly due to the multiple secretomes secreted from transplanted DPSCs or directly injected DPSC-SFs in the early phase of transplantation/administration.

## 1. Introduction

Diabetic polyneuropathy has high morbidity and negatively affects the quality of life among patients with diabetes [1]. Several randomized controlled trials revealed that good glycemic control is important to reduce the prevalence of diabetic polyneuropathy [2,3,4]. However, the Steno type 2 randomized study (STENO-2 study) demonstrated that better glycemic control is not enough to prevent diabetic polyneuropathy [5]. The impairments of nerve cells (such as neurons and Schwann cells) and vascular cells are primarily involved in the development of diabetic polyneuropathy [6]. Other factors, such as aging and chronic inflammation, also affect diabetic polyneuropathy. However, current treatments are mainly symptomatic treatments for pain. There is a demand for treatments based on the pathogenesis of diabetic polyneuropathy. Regenerative medicine is expected as a novel therapy for diabetic polyneuropathy.

We and others have demonstrated the effects of stem cell transplantation on diabetic polyneuropathy [7,8,9]. The transplantation of stem cells, such as bone marrow-derived mesenchymal stem cells, embryonic stem (ES) cell/induced pluripotent stem (iPS) cell-derived cells, and dental pulp stem cells (DPSCs), into the hindlimb skeletal muscles ameliorated the impaired nerve conduction velocity and nerve blood flow, as well as increased the capillary/muscle ratio, in diabetic animals [7,10,11,12]. We transplanted these stem cells into the unilateral hindlimb skeletal muscles, and the effects of transplantation were observed in the transplanted sides of the diabetic animals. DPSCs, a type of mesenchymal stem cell located in the tooth cavity, are attractive stem cell sources because DPSCs can be isolated from extracted teeth for orthodontic reasons without further invasion and cryopreserved while retaining viability [11]. Since orthodontic extractions are performed at a young age in many cases, we can easily obtain young DPSCs, which may overcome stem cell dysfunction due to aging or diseases. Using streptozotocin (STZ)-induced diabetic models, we confirmed that the transplantation of DPSCs ameliorated diabetic polyneuropathy [11,13,14].

Stem cell transplantation has been widely investigated as a treatment for many diseases, including ischemic cardiovascular disease and brain infarction, and successful results were reported [15,16]. However, previous investigations demonstrated that most of the transplanted stem cells disappeared from the transplanted site after transplantation [17,18]. We also confirmed that only a small number of the transplanted stem cells remained at the transplanted site 4 weeks after transplantation [11]. Therefore, the therapeutic mechanisms of stem cell transplantation are considered to be associated with the abundant secretomes from transplanted stem cells in the early phase of transplantation.

We already demonstrated that the administration of secreted factors from DPSCs (DPSC-SFs) could improve nerve conduction velocity and nerve blood flow in the diabetic rats [19]. However, it is not clear which is more effective for treating diabetic polyneuropathy between stem cell transplantation and the administration of secreted factors. In this study, we directly compare the effects of the transplantation of DPSCs and the administration of DPSC-SFs on diabetic polyneuropathy.

## 2. Results

### 2.1. Blood Glucose Levels and Body Weights

**Figure 1:**
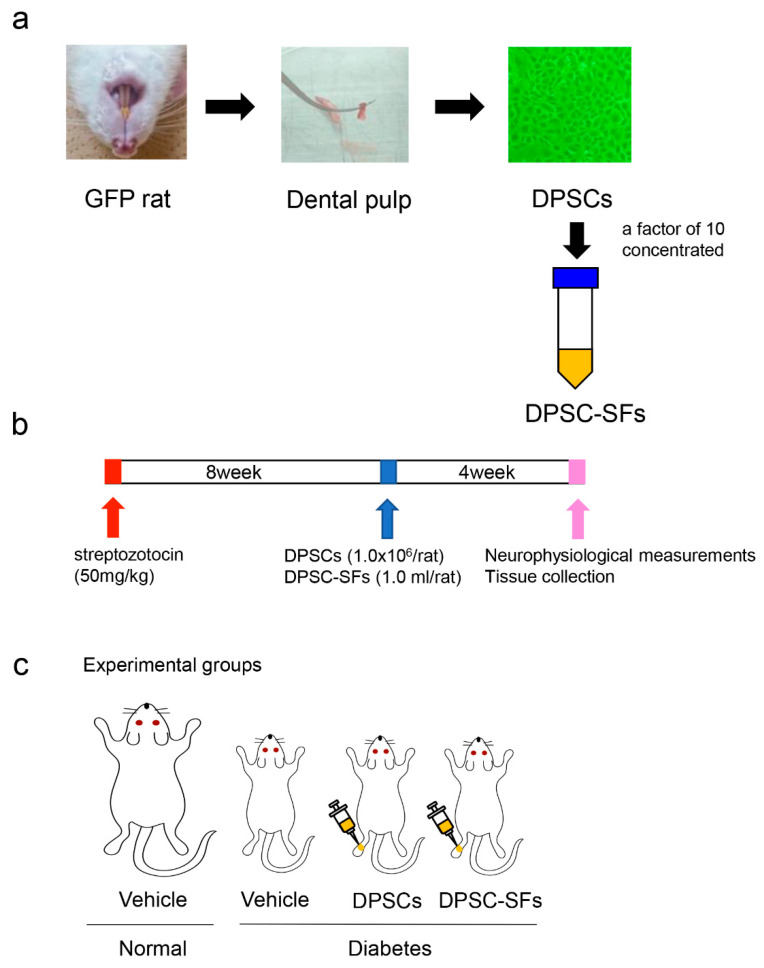
Experimental protocol. (**a**) Preparation of dental pulp stem cells (DPSCs) and secretory factors from dental pulp stem cells (DPSC-SFs). DPSCs were isolated from extracted dental pulp and cultured in an alpha modification of Eagle’s medium (α-MEM). The culture medium was collected after a 24-h culture of DPSCs in the serum-free medium and concentrated by a factor of 10 using 3-kDa centrifugal filters as secreted factors from the DPSCs (DPSC-SFs). (**b**) Animal experimental protocol. Eight weeks after the streptozotocin injection, DPSCs (1 × 10^6^ cells/rat) or DPSC-SFs (1.0 mL/rat) were administered. Neurophysiological measurements and tissue collection were performed 4 weeks after the administration of DPSCs/DPSC-SFs. (**c**) Experimental groups. There were four groups: normal + vehicle group, diabetes + vehicle group, diabetes + DPSCs group, and diabetes + DPSC-SFs group. DPSCs or DPSC-SFs were administered into the unilateral hindlimb skeletal muscles of the diabetic rats.

**Figure 2:**
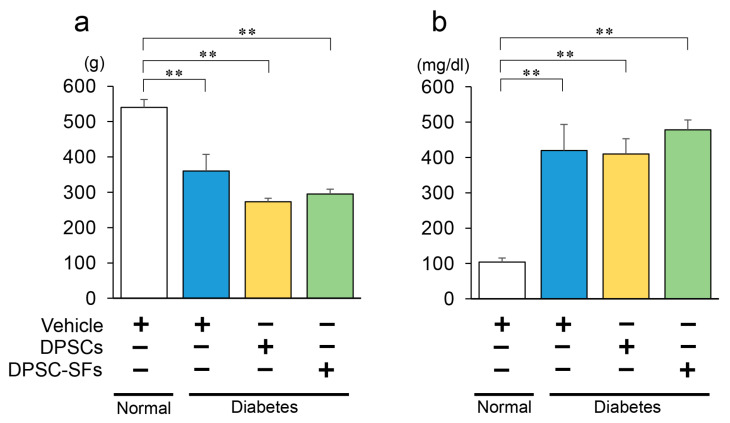
Body weights and blood glucose levels 4 weeks after DPSC/DPSC-SF administration. (**a**) Body weights (*n* = 6–7) and (**b**) blood glucose levels (*n* = 6–7). The results are presented as the mean ± SEM. ** *p* < 0.01.

The experimental protocol is depicted in Figure 1. Four weeks after the administration of DPSCs/DPSC-SFs, the body weights (Figure 2a) and blood glucose levels (Figure 2b) were measured. The diabetic rats showed significantly lower body weights and higher blood glucose levels compared to the normal rats (*p* < 0.01). Both DPSC transplantation and DPSC-SF administration did not significantly change the body weights and blood glucose levels of the diabetic rats (body weights: diabetes + vehicle 360.0 ± 47.4 g, diabetes + DPSCs 273.3 ± 9.5 g, diabetes + DPSC-SFs 295.0 ± 13.6 g, Blood glucose: diabetes + vehicle 419.4 ± 74.0 mg/dL, diabetes + DPSCs 409.8 ± 42.9 mg/dL, diabetes + DPSC-SFs 478.3 ± 27.9 mg/dL).

### 2.2. Comparison between DPSC Transplantation and DPSC-SF Administration on Sciatic Motor Nerve Conduction Velocity (MNCV), Sciatic Sensory Nerve Conduction Velocity (SNCV), and Sciatic Nerve Blood Flow (SNBF)

**Figure 3:**
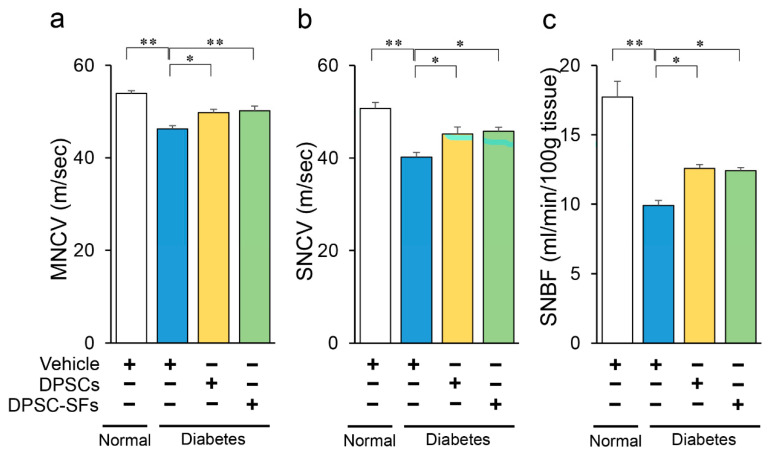
Effects of DPSC transplantation and DPSC-SF administration on sciatic motor/sensory nerve conduction velocity (MNCV/SNCV) and sciatic nerve blood flow (SNBF). (**a**) MNCV was measured between the ankle and sciatic notch (*n* = 6–7). (**b**) SNCV was measured between the ankle and knee (*n* = 5–7). (**c**) SNBF was measured using a laser Doppler blood flow meter (*n* = 6–7). All results are presented as the mean ± SEM, * *p* < 0.05, ** *p* < 0.01.

DPSC transplantation or DPSC-SF administration were performed eight weeks after STZ injection. DPSCs and DPSC-SFs were administered to 10 points of the unilateral hindlimb skeletal muscles of the diabetic rats, and a vehicle was injected into the contralateral hindlimb skeletal muscles. Four weeks later, the MNCV, SNCV, and SNBF were measured (Figure 3a–c). The MNCV, SNCV, and SNBF were significantly diminished among the diabetic rats. Both DPSC transplantation and DPSC-SF administration significantly ameliorated impaired MNCV, SNCV, and SNBF in the diabetic rats, but there were no significant differences in the improvement effects between DPSC transplantation and DPSC-SF administration (MNCV: diabetes + DPSCs 49.8 ± 0.8, diabetes + DPSC-SFs 50.2 ± 1.0 m/s, SNCV: diabetes + DPSCs 45.2 ± 1.5, diabetes + DPSC-SFs 45.8 ± 0.8 m/s, SNBF: diabetes + DPSCs 12.6 ± 0.3, diabetes + DPSC-SFs 12.4 ± 0.2 mL/min/100 g tissue).

Next, we compared the MNCV, SNCV, and SNBF on the DPSC/DPSC-SF-administered side and the contralateral side in the diabetes + DPSCs rats and the diabetes + DPSC-SF rats. Both DPSC transplantation and DPSC-SFs administration significantly increased MNCV, SNCV, and SNBF compared with the values on the contralateral sides of the diabetic rats, demonstrating that the efficacies of DPSC transplantation and DPSC-SF administration were limited to the administration site (data not shown).

### 2.3. Effects of DPSC Transplantation and DPSC-SF Administration on Intraepidermal Nerve Fiber Density (IENFD)

**Figure 4:**
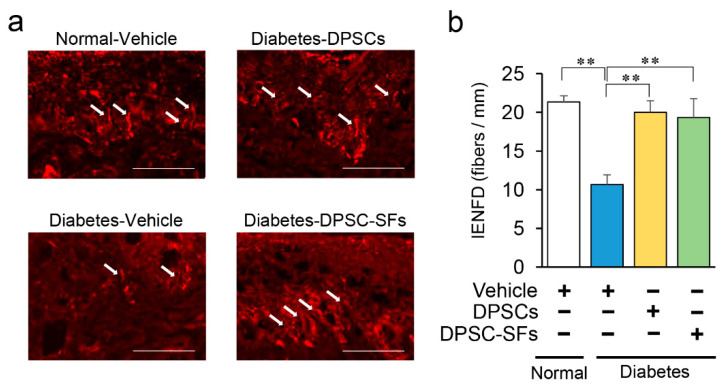
Effects of DPSC transplantation and DPSC-SF administration on the intraepidermal nerve fiber densities (IENFDs). (**a**) Representative images of intraepidermal nerve fiber profiles. Nerve fibers were visualized by staining with anti-PGP9.5 antibody. White arrows demonstrated unmyelinated small fibers in epidermis. Bar = 50 µm. (**b**) IENFD was counted under a confocal system (*n* = 5). Results are presented as the mean ± SEM, ** *p* < 0.01.

Since diabetic polyneuropathy is initially impaired by small fibers of the peripheral nerves, we evaluated IENFD in the footpads (Figure 4a). IENFD was reduced by 50% in diabetes + vehicle rats compared with normal + vehicle rats (*p* < 0.01), indicating the presence of small sensory nerve fiber degeneration (Figure 4b). Both DPSC transplantation and DPSC-SF administration significantly increased IENFD in the diabetic rats by 88% and 81%, respectively, four weeks after transplantation/administration (*p* < 0.01). There was no significant difference between DPSC transplantation and DPSC-SF administration.

### 2.4. Effects of DPSC Transplantation and DPSC-SF Administration on the Hindlimb Skeletal Muscles

**Figure 5:**
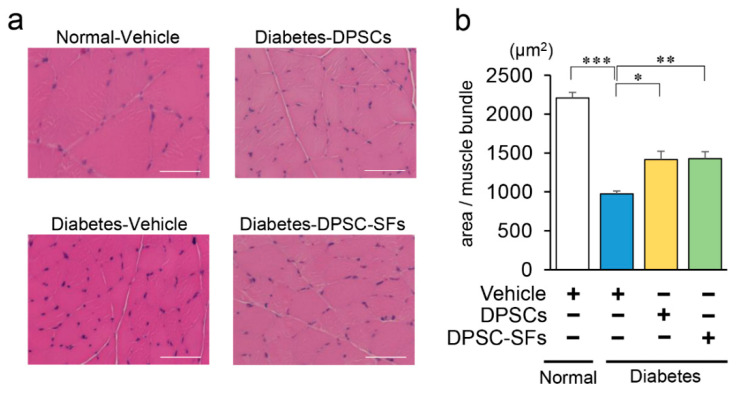
Effects of DPSC transplantation and DPSC-SF administration on the morphology of the hindlimb skeletal muscles. (**a**) Representative images of gastrocnemius muscles. Bar = 50 µm. (**b**) The size of muscle bundles was measured by ImageJ (*n* = 5). Results are presented as the mean ± SEM, * *p* < 0.05, ** *p* < 0.01, *** *p* < 0.001.

To confirm the morphological and pathological changes of hindlimb skeletal muscles, we measured the size of muscle bundles in the gastrocnemius muscles (Figure 5a,b). In diabetes + vehicle rats, the average size per muscle bundle was reduced to 44% compared with that in control + vehicle rats (*p* < 0.001). DPSC transplantation and DPSC-SF administration both significantly increased the size of muscle bundles by 1.5-fold compared with that of diabetes + vehicle rats (*p* < 0.05, *p* < 0.01, respectively). The sizes of muscle bundles were not significantly different between diabetes + DPSCs rats and diabetes + DPSC-SFs rats.

**Figure 6:**
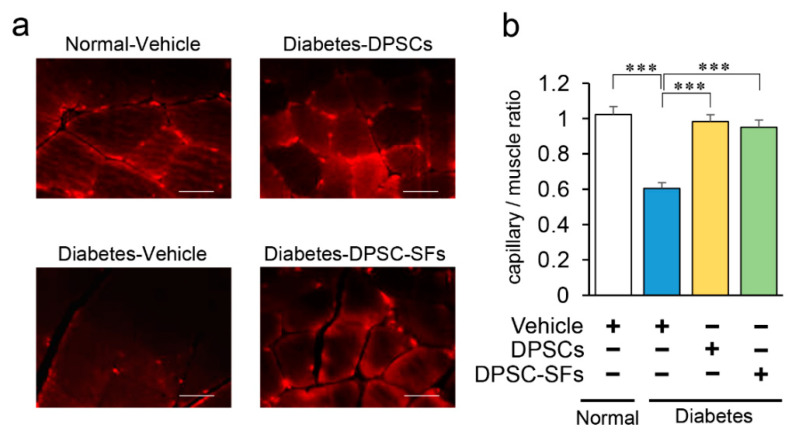
Effects of DPSC transplantation and DPSC-SF administration on the capillary density in the hindlimb skeletal muscles. (**a**) Representative photomicrographs of immunohistological staining of the gastrocnemius muscles. Capillaries were visualized with platelet endothelial cell adhesion molecule-1. Bar = 25 µm. (**b**) Quantitative analysis of the capillary/muscle bundle ratio (*n* = 5). Results are presented as the mean ± SEM, *** *p* < 0.001.

Next, we evaluated capillary number in the gastrocnemius muscles. Capillaries were visualized by the staining of vascular endothelial cells by anti-platelet endothelial cell adhesion molecule-1 (PECAM-1) antibody. As shown in Figure 6a and 6b, the capillary/muscle ratio was significantly decreased in the diabetes + vehicle rats by 41% (*p* < 0.001). DPSC transplantation and DPSC-SF administration ameliorated the reduction of capillary density in the muscles of the diabetic rats by 90% and 82%, respectively (*p* < 0.001). There was no significant difference in the efficacy on capillary/muscle ratio between DPSC transplantation and DPSC-SF administration.

### 2.5. Effects of DPSC Transplantation and DPSC-SF Administration on mRNA Expressions of Neurotrophic Factors in the Hindlimb Skeletal Muscles

**Figure 7:**
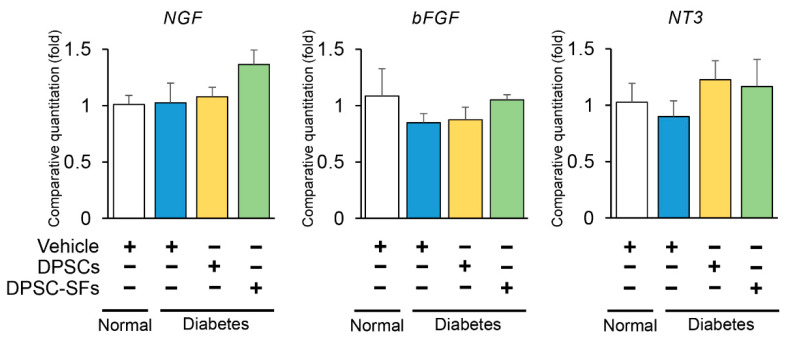
Effects of DPSC transplantation and DPSC-SF administration on mRNA expressions of nerve growth factor (NGF), basic fibroblast growth factor (bFGF), and neurotrophin-3 (NT-3) in the hindlimb skeletal muscles. The mRNA levels of these factors were evaluated by real-time PCR. The relative quantity was calculated by the ΔΔCt method using β2 microglobulin as the endogenous control. Results are expressed as the means ± SEM (*n* = 4–6).

Since DPSCs expressed neurotrophic factors, gene expressions of the gastrocnemius muscles were evaluated by real-time PCR 4 weeks after DPSC/DPSC-SF administration. As shown in Figure 7, there was a tendency towards a decrease in basic fibroblast growth factor (bFGF) and neurotrophin-3 (NT-3) mRNA expressions in the diabetes + vehicle rats and an increase in nerve growth factor (NGF), bFGF, and NT-3 mRNA expressions in the diabetes + DPSC-SFs rats and an increase in NT-3 mRNA expression in the diabetes + DPSCs rats. However, all of these were not significant. Neither DPSC transplantation nor DPSC-SF administration significantly affected these gene expressions.

### 2.6. Characterization of Secreted Factors from Dental Pulp Stem Cells

We previously investigated the gene expressions of DPSCs and demonstrated multiple gene expressions, such as angiogenic (e.g., vascular endothelial growth factor (VEGF), bFGF), neurotrophic (e.g., NGF, NT-3), and immunosuppressive factors (e.g., macrophage colony stimulating factor (M-CSF)) [13]. To characterize the profile of DPSC-SFs, we analyzed 90 rat proteins using rat antibody array (RayBiotech, Inc. Peachtree Corners, GA, USA).

**Figure 8:**
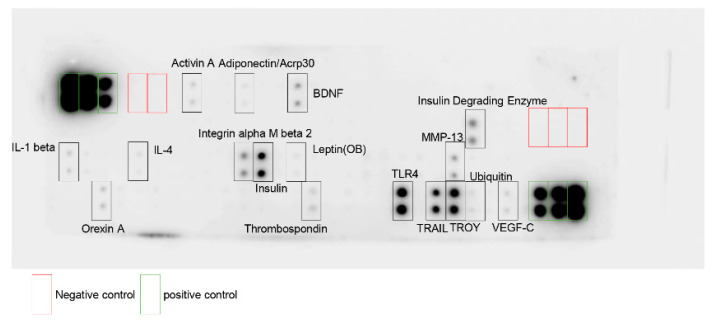
Evaluation of DPSC-SFs using rat antibody array (RayBiotech). Representative image of dot blot is shown. Main dot is surrounded by a square and shows the name of the protein. BDNF, brain-derived neurotrophic factor; IL-1 beta, interleukin-1 beta; IL-4, interleukin-4; MMP-13, matrix metalloproteinase-13; TLR4, toll-like receptor 4; TRAIL, tumor necrosis factor-related apoptosis-inducing ligand; TROY, tumor necrosis factor receptor superfamily, membrane 19.

As shown in Figure 8, DPSC-SFs included more than 17 proteins from the investigated 90 proteins. We could observe angiogenic factors (e.g., VEGF-C), neurotrophic factors (e.g., brain-derived neurotrophic factor (BDNF)), and immunomodulative factors (e.g., interleukin-1 beta (IL-1 β), interleukin-4 (IL-4), toll-like receptor 4 (TLR4)), as well as other multifunctional factors.

## 3. Discussion

In this study, we performed a direct comparison of the therapeutic effects of DPSC transplantation and DPSC-SF administration on diabetic polyneuropathy. The diabetic rats demonstrated decreased sciatic motor/sensory nerve conduction velocity and sciatic nerve blood flow compared to normal rats. Both DPSC transplantation and DPSC-SF administration into the hindlimb skeletal muscles ameliorated impaired MNCV, SNCV, and SNBF in the diabetic rats, and the degrees of improvement were almost the same between these two different therapies.

Nerve conduction velocity is considered as a gold standard method to evaluate diabetic polyneuropathy because it is reliable and reproducible [20]. So far, there are few drugs to improve nerve conduction velocity in patients with diabetic polyneuropathy. Aldose reductase inhibitor is a drug to improve nerve conduction velocity. A three-year administration of aldose reductase inhibitor significantly improved motor nerve conduction velocity only in patients with HbA1c below 7% [21]. In this study, we demonstrated that DPSC transplantation improved motor nerve conduction velocity by 3.5 m/s and sensory nerve conduction velocity by 5.0 m/s, and DPSC-SF administration improved motor nerve conduction velocity by 3.9 m/s and sensory nerve conduction velocity by 5.6 m/s, without the improvement of glycemic control.

In diabetes, the dysfunction of vascular cells and nerve cells caused by hyperglycemia results in impaired nerve blood flow and nerve dysfunction in the peripheral nerves; in this way, diabetic polyneuropathy develops and progresses [6]. Other factors, including aging, chronic inflammation, and hyperlipidemia, are exacerbating factors in diabetic polyneuropathy [1,22]. In peripheral nerves with diabetic polyneuropathy, disorders such as ischemia, axonal degeneration, Schwann cell–axon transport disorder, demyelination, and impairments of neurofilament synthesis occur [6].

Previously studied regenerative medicine for diabetic polyneuropathy mainly included stem cell transplantation therapies. The therapeutic mechanisms of stem cell transplantation on diabetic polyneuropathy are the following: (1) angiogenesis and improvement of blood flow, (2) anti-inflammatory action, and (3) neuroprotective action [7,11,13,14,23,24]. On the other hand, in related studies, most grafted progenitor/stem cells consistently disappeared sometime after transplantation [11,18,25]. Currently, the therapeutic mechanism of progenitor/stem cell transplantation is considered to be mainly associated with the abundant secretomes of transplanted stem cells in the early phase of transplantation. According to the hypothesis of secretomes, we investigated the effects of DPSC-SF administration on diabetic polyneuropathy [19]. We confirmed that DPSC-SF administration ameliorates sciatic nerve conduction velocity, sciatic nerve blood flow, and intraepidermal nerve fiber density, as well as the capillary/muscle bundle ratio, in the skeletal muscles. An in vitro study revealed that DPSC-SFs promote the neurite outgrowth of dorsal root ganglion cells, the proliferation of Schwann cells, and myelin protein formation [14].

In this study, both DPSC transplantation and DPSC-SF administration showed almost the same level of efficacy on the small fiber degeneration and capillary density in the hindlimb skeletal muscles. We also demonstrated that DPSC transplantation and DPSC-SF administration ameliorated skeletal muscle atrophy in the diabetic rats almost equally. DPSCs expressed multifunctional genes including angiogenic, neurotrophic, and immunomodulative genes [13]. The investigation of human DPSC gene profiles using DNA microarray demonstrated multiple gene expressions which showed a multipotent ability proliferate or differentiate, or cytokines and others [26]. We confirmed that DPSC-SFs contained angiogenic, neurotrophic, and immunomodulative proteins. A direct comparison between DPSC transplantation and DPSC-SF administration revealed that the efficacy of both treatments on diabetic polyneuropathy was almost the same at least one month after administration. Furthermore, since DPSCs expressed neurotrophic factors, we observed whether the expression of these neurotrophic factors was increased in the DPSC/DPSC-SF administration site. Although we hypothesized that these neurotrophic factors increased in DPSC-transplanted skeletal muscles, there were no significant differences between DPSC transplantation and DPSC-SF administration. These results suggest that the efficacy of both DPSC transplantation and DPSC-SF administration for diabetic polyneuropathy four weeks after transplantation/administration was mainly due to the multiple secretomes secreted from transplanted DPSCs or directly injected DPSC-SFs in the early phase of transplantation/administration.

Although we can avoid stem cell dysfunction due to aging and diabetes by obtaining DPSCs at a young age and keeping them until needed, there are still several risks we need to consider for DPSC transplantation. Tumorigenesis of the transplanted stem cells was rarely reported. However, we still have to observe very carefully after transplantation [27]. In the case of an allograft, immunosuppressive agents need to be administered continuously to avoid graft versus host disease. DPSC-SF administration may resolve these risks. In addition, we should select high-quality DPSCs and obtain an abundance of high-viability DPSC-SFs, which may lead to cost reductions. On the other hand, since at least some of the transplanted DPSCs were retained at the transplant site and some were differentiated into vascular endothelial-like cells, the duration of efficacy might be longer in DPSC transplantation than DPSC-SF administration. Further study is required on this issue.

In conclusion, a direct comparison revealed that both DPSC transplantation and DPSC-SF administration into the skeletal muscles ameliorated diabetic polyneuropathy with very similar effects. However, there are several differences in the benefits and risks between them. We require more investigations to determine which therapy should be used in each case.

## 4. Materials and Methods

### 4.1. Animals

Male Sprague–Dawley (SD) rats were obtained from Chubu Kagakushizai (Nagoya, Japan) at 6 weeks of age. STZ (Wako, Osaka, Japan; 50 mg/kg bodyweight in 0.9% sterile saline) was injected intraperitoneally to induce diabetes. Rats with a blood glucose level > 14 mmol/L were used as diabetic animals. This study was approved by the Institutional Animal Care and Use Committees of Aichi Gakuin University (AGUD 318) on December 18th, 2015, and all animal experiments were carried out following national guidelines and the relevant national laws on the protection of animals.

### 4.2. Isolation and Culture of Rat DPSCs

Dental pulp was harvested from the extracted incisors of 6-week-old male green fluorescent protein (GFP)-transgenic SD rats (SD-Tg(CAG-EGFP)Cz-0040sb) (Japan SLC, Inc., Hamamatsu, Japan) and suspended in phosphate-buffered saline containing 0.1% collagenase and 0.25% trypsin- ethylenediaminetetraacetic acid (EDTA). DPSCs were cultured in an alpha modification of Eagle’s medium (α-MEM) (GIBCO, Thermo Fisher Scientific, Waltham, MA, USA) with 20% fetal bovine serum (GIBCO) on plastic dishes at 37 °C in 5% humidified CO_2_. Non-adherent cells were washed off, and adherent cells were continuously expanded until the third passage. DPSCs expressed the cell surface markers of mesenchymal stem cells, such as CD29 and CD90, and their pluripotency to chondrocytes, adipocytes, and osteoblasts was confirmed [13].

### 4.3. Preparation of DPSC-SFs

When cultured DPSCs reached confluence in a 10-cm culture dish, the number of DPSCs was around 1 × 10^6^ cells/dish. The culture medium was collected after a 24-h culture of DPSCs in serum-free medium and concentrated by a factor of 10 using 3-kDa centrifugal filters (Amicon Ultra-15; Nihon Millipore, Tokyo, Japan) as secreted factors from DPSCs (DPSC-SFs).

### 4.4. Transplantation of DPSCs and Administration of DPSC-SFs

We divided the diabetic rats into three groups: the diabetes + vehicle group, the diabetes + DPSCs group, and the diabetes + DPSC-SFs group. Eight weeks after STZ injection, a DPSC suspension (1.0 mL in total, 1 × 10^6^ cells) was injected at 10 points into the unilateral hindlimb skeletal muscles in the diabetes + DPSCs rats. DPSC-SFs (1.0 mL/rat) were administered at 10 points in the unilateral hindlimb skeletal muscles in the diabetes + DPSC-SFs rats. Four weeks after administration, the following measurements were carried out.

### 4.5. Sciatic Motor and Sensory Nerve Conduction Velocities

Rats were anesthetized with isoflurane and placed on a heated pad in a room maintained at 25 °C, and the near-nerve temperature was maintained at 37 °C using a BAT-12 multipurpose thermometer (Bioresearch Co. Nagoya, Japan). MNCV between the ankle and the sciatic notch and SNCV between the ankle and the knee were measured by a Neuropak MEB-9400 instrument (Nihon-Koden, Osaka, Japan) as described previously [19].

### 4.6. Sciatic Nerve Blood Flow

Rats were placed on a warming pad and anesthetized by isoflurane. The femur skin was cut, and a laser probe was placed just above the exposed sciatic nerve. SNBF was measured using a laser Doppler blood flow meter (FLO-N1; Omega Wave Inc., Tokyo, Japan).

### 4.7. Intraepidremal Nerve Fiber Density

Rats were killed by CO_2_ inhalation. Plantar skin was removed. After fixation, longitudinal 25-mm thick footpad sections from each rat were cut on a cryostat (Leica Microsystems, Wetzlar, Germany). The sections were incubated with anti-PGP9.5 antibody (Millipore, Tokyo, Japan). Alexa Fluor 555-coupled goat anti-mouse immunoglobulin G antibody (Invitrogen, Carlsbad, CA, USA) was applied as the secondary antibody. Nerve fibers were counted under an FV10i confocal system (Olympus, Tokyo, Japan).

### 4.8. Histological Analysis and Immunohistological Staining

Paraffin-embedded gastrocnemius muscles were cut into 5-μm sections for histological analysis and immunohistochemical staining. Sections were subjected to hematoxylin and eosin (HE) staining to measure the area of the muscle bundles. For the detection of vascular endothelial cells in the gastrocnemius muscles, the sections were incubated with anti-PECAM-1 monoclonal antibody (Dianova, Hamburg, Germany) and stained using an Alexa Fluor 555-coupled goat anti-mouse immunoglobulin G antibody (Invitrogen). Slides were observed under light and fluorescence microscopes.

### 4.9. mRNA Expression Analyses

Gastrocnemius muscles were removed and immersed in an RNAlater ribonucleic acid stabilization reagent (QIAGEN, Hilden, Germany). RNA was extracted by an RNeasy Mini Kit (QIAGEN) according to the manufacturer’s instructions. The cDNA was synthesized using ReverTra Ace (Toyobo, Osaka, Japan). TaqMan Gene Expression Assay primers and probes for NGF, bFGF, and NT-3 were purchased from Applied Biosystems (Foster City, CA, USA). Real-time quantitative PCR was performed and monitored by a LightCyclerR 480 Instrument II (Roche, Basel, Switzerland). The relative quantity was calculated by the ΔΔCt method using β2 microglobulin as the endogenous control.

### 4.10. Analysis of Proteins in DPSC-SFs

To determine proteins in DPSC-SFs, DPSC-SFs were analyzed using L-Series rat antibody array 90 (Ray Biothech Inc., Peachtree Corners, GA, USA) according to the manufacturer’s instructions. Biotin-labeled DPSC-SFs were added onto the membrane and incubated for 1 h. After washing, horseradish peroxidase-conjugated streptavidin was added to the membrane. The membrane was visualized by a Chemidoc Touch (Bio-rad, Hercules, CA, USA) and the data were analyzed using ImageJ software.

### 4.11. Statistical Analysis

Data were processed using GraphPad Prism (GraphPad Software, San Diego, CA, USA) and are represented as the mean ± SEM. Statistical significance was analyzed by a one-way ANOVA, Bonferroni correction for multiple comparisons. Differences at *p* < 0.05 were considered significant.

## Abbreviations

BDNF	Brain-derived neurotrophic factor
bFGF	Basic fibroblast growth factor
DPSC	Dental pulp stem cell
DPSC-SF	Dental pulp stem cell-secreted factors
EDTA	Ethylenediaminetetraacetic acid
ES	Embryonic stem
GFP	Green fluorescent protein
iPS	Induced pluripotent stem
M-CSF	Macrophage colony-stimulating factor
MEM	Minimum essential medium
MNCV	Motor nerve conduction velocity
NGF	Nerve growth factor
NT-3	Neurotrophin-3
PECAM	Platelet endothelial cell adhesion molecule
SD	Sprague–Dawley
SNBF	Sciatic nerve blood flow
SNCV	Sensory nerve conduction velocity
STZ	Streptozotocin
VEGF	Vascular endothelial growth factor
